# Pressure Anaesthesia

**Published:** 1907-04

**Authors:** Gustavus North

**Affiliations:** Cedar Rapids, Iowa.


### PRESSURE ANAESTHESIA.

BY DR. GUSTAVUS NORTH, Cedar Rapids, Iowa.

A prominent dentist asked me the question a short time ago; "What is the greatest discovery in dentistry the past few years?" I readily answered; "Pressure Anæsthesia for the treatment of sensitive dentine and removing pulps."

I will not give the modus operandi of pressure anæsthesia in this short article, as most dentists are familiar with it.

I believe pressure anæsthesia is the most wonderful discovery that has come before the dental profession in my forty years practice.

Most all operations upon the teeth can be performed with out pain by the use of neurocain or cocain.

Sensitive cavities can be prepared and filled, pulps removed, cavities filled or crowned at the same setting, with out pain by the use of pressure anæsthesia, with out any discomfort following.

I told the students years ago that the coming dentist would be the man that could perform operations without pain.

